# Concurrent Persistent Truncus Arteriosus and Left Atrial Diverticulum in a Domestic Short-Haired Cat

**DOI:** 10.3390/ani15060899

**Published:** 2025-03-20

**Authors:** Irina Constantin, Alexandra Cofaru, Raluca Murariu, Iuliu Călin Scurtu, Flaviu-Alexandru Tăbăran

**Affiliations:** 1 Department of Pathology, University of Agricultural Sciences and Veterinary Medicine, 400372 Cluj-Napoca, Romania; alexandru.tabaran@usamvcluj.ro; 2 Department of Small Animal Internal Medicine, University of Agricultural Sciences and Veterinary Medicine, 400372 Cluj-Napoca, Romania; alexandra.cofaru@usamvcluj.ro (A.C.); raluca.murariu@usamvcluj.ro (R.M.); iuliu.scurtu@usamvcluj.ro (I.C.S.)

**Keywords:** persistent truncus arteriosus, left atrial diverticulum, myxomatous degeneration, splenopancreatic fusion, feline

## Abstract

A 2-year-3-month-old neutered male domestic shorthair cat was presented for cardiological evaluation at the University of Agricultural Sciences and Veterinary Medicine in Cluj-Napoca, Romania, with a history of dyspnea, open-mouth breathing, cyanosis, and exercise intolerance. Physical examination identified a grade 4/6 systolic murmur, cyanosis, and mild dyspnea, with no abdominal abnormalities. Echocardiography revealed right ventricular hypertrophy, severe right atrial dilation, a thickened tricuspid valve, and a large nonrestrictive ventricular septal defect with a left-to-right shunt, consistent with persistent truncus arteriosus (PTA), confirmed postmortem. During necropsy, in addition to the clinically confirmed diagnoses, bilateral myxomatous degeneration of the A-V valves, and associated anomalies such as an atrial diverticulum and splenopancreatic fusion were observed and confirmed histologically. This case emphasizes the clinical and morphological complexity of congenital heart defects in felines, highlighting the importance of advanced diagnostic and histopathological techniques for accurate diagnosis and characterization.

## Simple Summary

A 2-year-old male cat was brought to a veterinary hospital due to difficulty breathing, cyanosis (blue gums), and low tolerance for exercise. A heart murmur and other symptoms prompted advanced tests, revealing a rare and severe heart defect called persistent truncus arteriosus (PTA), along with other abnormalities in the heart and surrounding organs. After a progressive worsening of the clinical state, the cat died, and the diagnosis was confirmed postmortem, uncovering additional defects like valve degeneration and abnormal organ connections. This case demonstrates the complexity of congenital heart defects in cats and underscores the importance of advanced imaging and postmortem examinations for accurate diagnosis. Such insights can improve understanding and management of similar conditions, benefiting pet health and veterinary care.

## 1. Introduction

Persistent truncus arteriosus (PTA) is a rare congenital anomaly in cats characterized by the failure of the embryonic truncus arteriosus to separate into the pulmonary artery and aorta [1,2,3,4,5,6,7,8]. This results in a single arterial trunk, which is commonly accompanied by a ventricular septal defect [1]. The most common clinical signs include dyspnea or tachypnea, cyanosis of the mucous membranes and exercise intolerance [3,6]. A clear outcome is difficult to define, as only a few cases have been reported in cats [2,3,4,5,6]. Based on the degree of aortic-pulmonary fusion, the PTA is classified in 4 types: type 1—where the truncus arteriosus gives rise to both the ascending aorta and a single pulmonary trunk, type 2—where from the dorsal wall of the truncus arteriosus, the right and left pulmonary arteries emerge close together, type 3—where from either side of the truncus arteriosus, one or both pulmonary arteries emerge separately, type 4—where the pulmonary arteries are absent, with an apparent congenital absence of the sixth aortic arches, while the pulmonary circulation is maintained through the bronchial arteries [1]. While extensively studied in human medicine, reports of PTA in domestic cats remain scarce, complicating diagnosis and management [2,3,4,5,6,7,8].

A left atrial diverticulum (LAD) is a pouch-like structure that emerges as a broad-based outpouching from the left atrial wall, characterized by a saclike shape and a smooth contour. Recognized as an anatomical variant, LADs communicate directly with the left atrial lumen and are typically small, measuring less than 1 cm in size, though larger diverticula are occasionally observed. These structures often present with a lobulated contour and may exhibit internal trabeculations [9,10].

This case report describes the clinical, echocardiographic, and postmortem findings of PTA type 1 associated with LAD in a 2-year-3-month-old domestic shorthair cat. The report underscores the necessity of a multidisciplinary approach, incorporating advanced imaging and histological analysis, to elucidate the pathophysiology of complex congenital heart defects and their systemic effects in companion animals.

## 2. Detailed Case Description

### 2.1. History and Clinical Examination

A 2-year-3-month-old, indoor, neutered male domestic shorthair cat, weighing 3.3 kg, was presented to the Cardiology Department at the University of Agricultural Sciences and Veterinary Medicine in Cluj-Napoca, Romania, for a complete cardiological examination. The cat had a history of dyspnea, open-mouth breathing, exercise intolerance and cyanosis. The clinical signs were noticed by the owner since the cat was adopted and exacerbated in the last 6 months.

The physical examination identified a lean cat with 3/9 body condition score, normothermic (38.2 °C), with a heart rate of 180 bpm and normal arterial pulses. The respiratory pattern was abnormal, characterized by an increased respiratory rate (48/min) and mild dyspnea. Cyanosis was noted in the conjunctival and oral mucous membranes, worsening with restlessness. Thoracic auscultation revealed a grade 4/6 systolic parasternal murmur in both hemithoraxes, with normal respiratory sounds. Abdominal palpation showed no abnormalities, and the abdominal contour appeared normal. A blood workup was not conducted.

### 2.2. Echocardiographic Findings

Transthoracic echocardiography was performed using the Esaote MyLabX8 Vet ultrasound unit (Esaote; Genova, Italy) (equipped with a dedicated phased array probe for cats (P5-13) with continuous ECG monitoring. Measurements were performed from digitally stored images. The electrocardiogram (ECG) showed a normal sinus rhythm with an average rate of 190 bpm. The patient was placed in right and left lateral recumbency, with prior preparation by shaving the fur and applying echographic gel. Echocardiography was performed by a cardiology resident. No sedation was used due to the patient’s cooperation.

**Figure 1:**
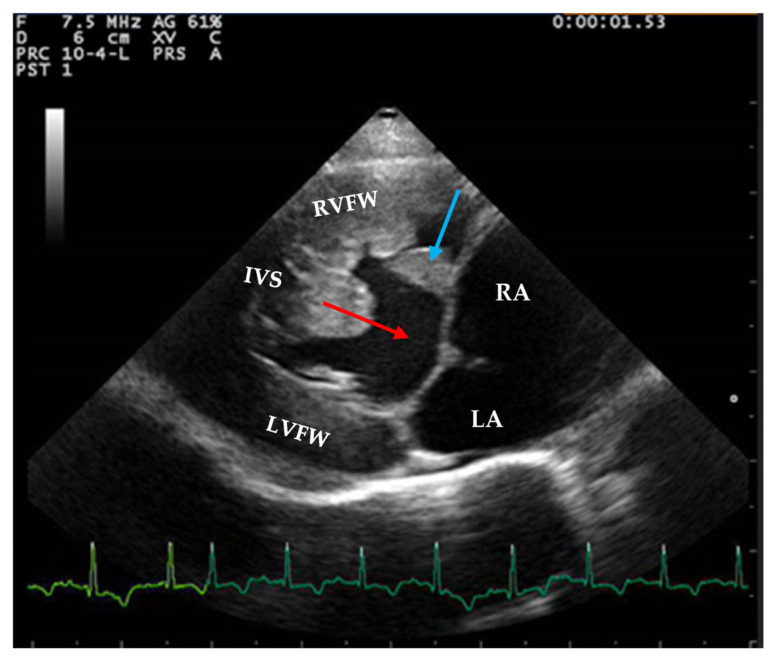
Right parasternal long axis view from a 2-year-3-month-old domestic shorthair neutered male cat. A large ventricular septal defect can be observed (red arrow); the tricuspid valve is severely thickened (blue arrow). Severe hypertrophy of the right ventricular free wall, moderate hypertrophy of the left ventricular wall, and right atrial dilation are present. RVFW—right ventricular free wall, IVS—interventricular septum, LVFW—left ventricular free wall, RA—right atrium, LA—left atrium.

**Figure 2:**
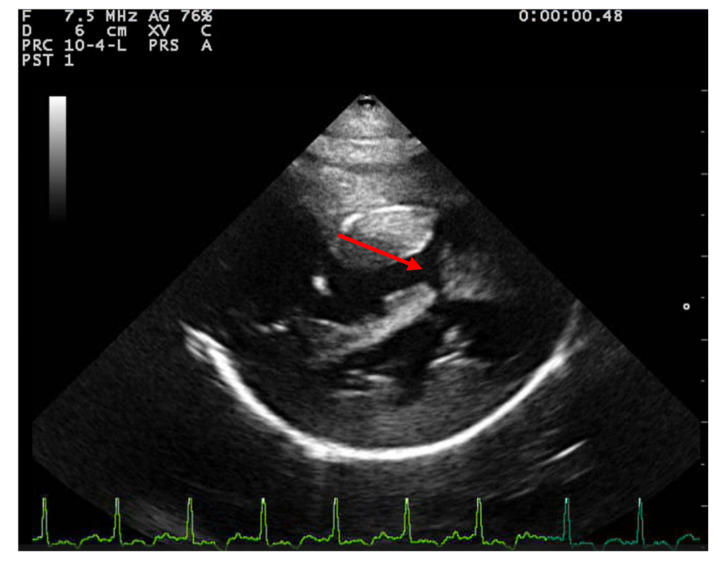
Right parasternal short axis view from a 2-year-3-month-old domestic shorthair neutered male cat underlying flattening of interventricular septum. The VSD can be observed as well (red arrow).

**Figure 3:**
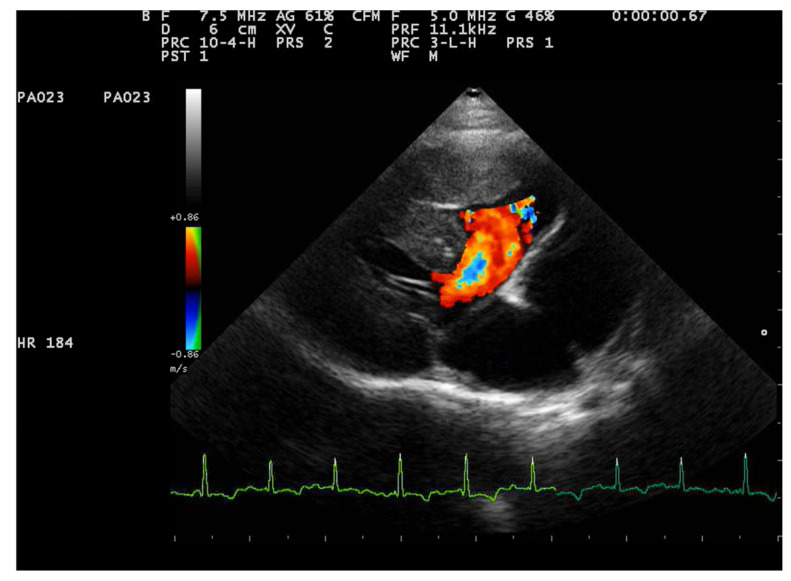
Right parasternal long axis view from a 2-year-3-month-old domestic shorthair neutered male cat. Color Doppler interrogation at the level of the ventricular sept defect identified a left-to-right flow.

Two-dimensional echocardiography revealed significant hypertrophy of the right ventricular free wall, with a diastolic thickness of 8.7 mm, accompanied by prominent papillary muscles. The right atrium was severely dilated (maximum diastolic cranio-caudal diameter of 20.3 mm, compared to the reference value of 12 mm in healthy cats) [11]. The leaflet edges of the tricuspid valve were severely thickened. (Figure 1). The left atrium was within normal limits, with a maximum diastolic cranio-caudal diameter of 13.7 mm, compared to the reference value of 16 mm [12], while the left ventricular free wall was hypertrophic, with a diastolic thickness of 7.3 mm measured in 2D echocardiography. In the right parasternal short-axis view at the level of papillary muscles, the interventricular septum was flattened in both systole and dyastole (Figure 2). A large interventricular septal defect (VSD) was identified in both right parasternal long-axis and short-axis views, with a diameter of at least 10.2 mm measured in the long-axis view. Color-flow Doppler examination revealed a left-to-right shunt through the VSD (Figure 3), with a peak velocity of 1.3 m/s. A single large trunk was identified, originating from the base of the heart and communicating with both ventricles. Caudal vena cava was not dilated and had an inspiratory collapse.

A contrast echocardiography was also performed which confirmed the presence of interventricular communication with bidirectional shunting.

### 2.3. Postmortem and Histological Findings

At the moment of diagnosis, no treatment was recommended, only a 3 month follow-up, which the owner did not pursue. The animal died 1 year and 9 months after the clinical diagnosis and a full necropsy was carried following a previously described technique focused on heart pathology [13]. For the examination of the heart, the previously described inflow-outflow technique was followed [13]. For a better assessment and preservation of the truncus arteriosus, the technique was modified, and the outflow incision was not performed for the right heart. During the necropsy, the heart, and samples of lung, spleen and pancreas were harvested. The tissues were fixed in 10% NBF, and after complete fixation, the gross examination was repeated before sectioning the tissues for routine paraffin embedding and final staining with hematoxylin and eosin [14].

**Figure 4:**
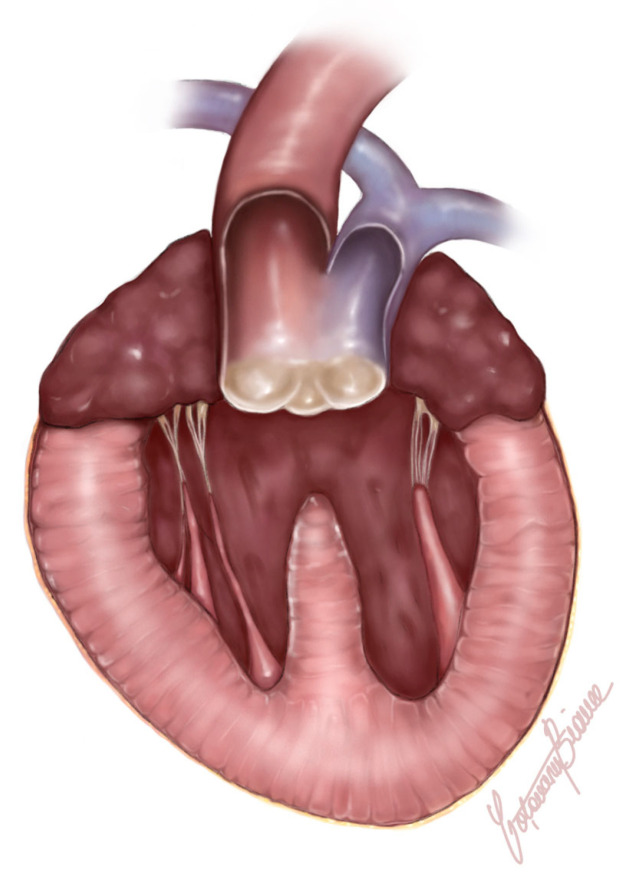
Schematic illustration depicting the gross anatomy of the truncus arteriosus in the presented case. Frontal view highlighting the abnormal fusion of the pulmonary artery and the aorta into a single arterial trunk arising from the ventricles, along with a prominent ventricular septal defect. Blue color coding (pulmonary artery) is used to differentiate the two blood vessels.

**Figure 5:**
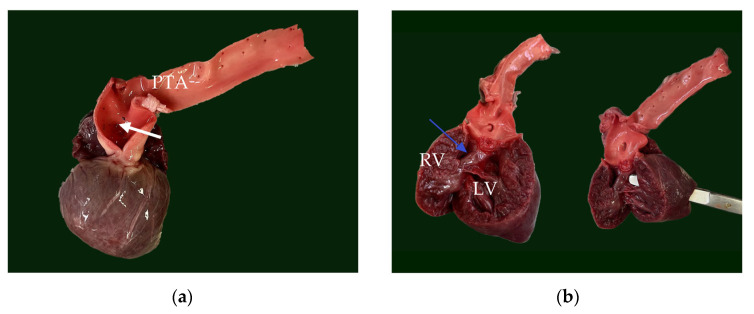

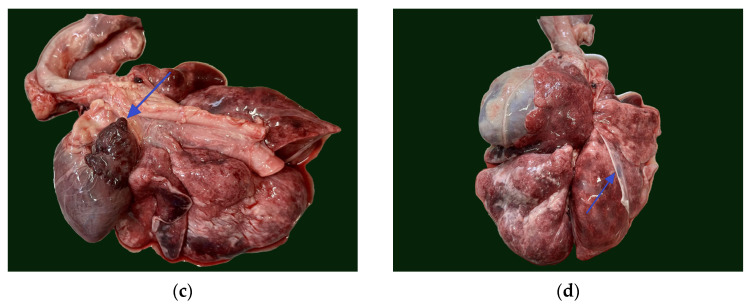
(**a**) Truncus arteriosus with the opening of pulmonary artery (white arrow). (**b**) The septal defect (blue arrow) which created direct communication between the two ventricles through the septal defect; the scissor could transverse directly from left ventricle to right ventricle. (**c**) Exterior aspect of the left atrial diverticulum (blue arrow) which originated from the left atrium. (**d**) The gross aspect of the abnormal fibrous membrane (blue arrow) adherent to lung pleura. PTA—persistent truncus arteriosus, RV—right ventricle, LV—left ventricle.

The heart was significantly enlarged (organ weighed 37 g, representing 1.12% of total body weight) with prominently thickened, ventricular walls (bilateral ventricular hypertrophy). Originating from the base of the heart, a single large arterial trunk (PTA) (measuring 0.8 cm) with its ventricular outflow tract was observed (Figure 4). At 0.5 cm from its origin, the PTA gave rise to the pulmonary artery, which subsequently branched into the right and left pulmonary arteries. Under the arterial trunk, within the upper segment of the interventricular septum a large and non-restrictive septal defect measuring 1.2 cm was observed. Due to all the identified features, the single large arterial trunk was identified as a persistent truncus arteriosus type 1 (Figure 5a,b).

**Figure 6:**
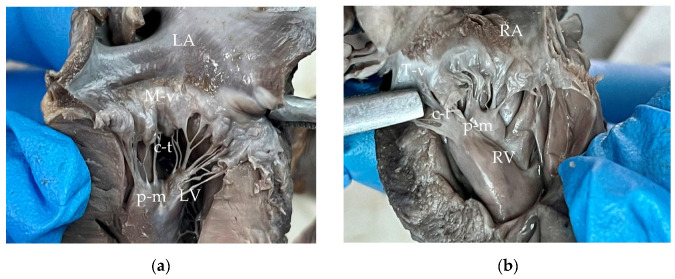
Gross appearance of formalin-fixed heart: (**a**) Left heart—mitral valve with an appearance characteristic to myxomatous degeneration, the valve appears thickened and nodular, and chordae tendineae were also thickened. (**b**) Right heart—tricuspid valve with an incipient stage of myxomatous degeneration, thickened chordae tendineae, supernumerary and hypertrophied papillary muscles. LA—left atrium, LV—left ventricle, M-v—mitral valve, c-t—chordae tendineae, p-m—papillary muscles, RA—right atrium, RV—right ventricle, T-v—tricuspid valve.

**Figure 7:**
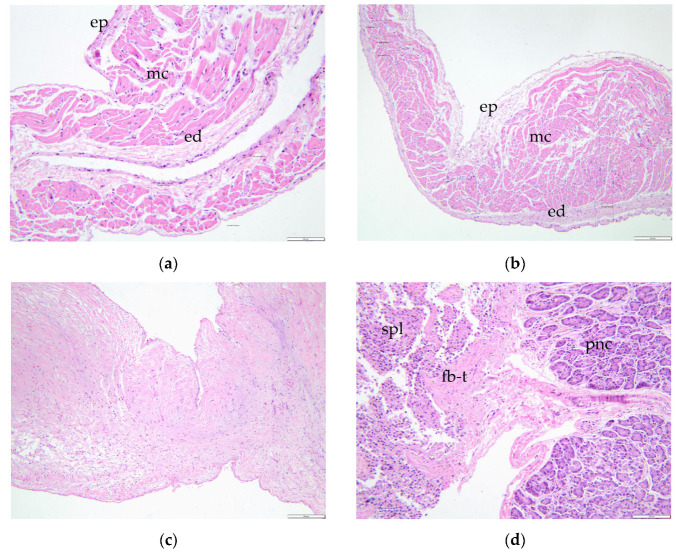
Histological features: (**a**) Left atrial divertculum presented a very thin aspect around its apex with a diameter reaching 136.56 μm, H&E stain, ob × 20. (**b**). Transition from the left atrial wall to the diverticulum, with thinning of the wall (from 936.26 μm to 304.97 μm), especially of the myocardium (from 771.78 μm to 190.01 μm) and endocardium (142.14 μm to 51.76 μm), H&E stain, ob × 10. (**c**) The mitral valve was presenting a myxomatous degeneration characteristic aspect, the spongiosa appeared thickened, while loosely arranged fibrous connective tissue were markedly disrupting and replacing the fibrosa, H&E stain, ob × 10. (**d**) Splenopancreatic fusion—the spleen and the pancreas are strongly bounded and intertwined through a fibrous component. The fibrous bridge spread through the splenic and pancreatic parenchyma, which conferred continuity between the two organs, H&E stain, ob × 20. A&D: Scale bar = 100 μm. B&C: Scale bar = 200 μm. ep—epicardium, mc—myocardium, ed—endocardium, spl—spleen, pnc—pancreas, fb-t—fibrous tissue.

The mitral valve was thickened and nodular and continued with thickened chordae tendineae (Figure 6a). The left atrium presented endocardial regurgitation scars (jet lines), as a consequence of mitral regurgitation. Within the left atrial auricle, a small outpouching structure (5 mm diameter) communicating to the atrial lumen was present (Figure 5c). This structure was identified as an atrial diverticulum within the confines of the pericardium. No signs of left atrial herniation or a pericardial defect were observed. The atrial diverticulum had a smooth interior wall and was completely covered by epicardium. Histologically, the diverticulum retained all three layers of the atrial wall, although it exhibited marked thinning of the myocardium and endocardium, with the thinnest area measuring less than 140 μm (Figure 7a,b).

Both the tricuspid valve and its chordae tendineae were diffusely thickened, accompanied by abnormal segmentation of the hypertrophied papillary muscles. The anterior papillary muscles measured 6 mm in diameter and featured a small appendage posteriorly. The posterior papillary muscles displayed a bifid appearance, while the sub arterial papillary muscles were subdivided into five distinct segments. These findings were consistent with the presence of supernumerary papillary muscles (Figure 6b).

In addition to the cardiac abnormalities, the evaluation of the thoracic cavity revealed a pulmonary pleural anomaly characterized by an abnormal pleural insertion at the level of the right middle lung lobe. This abnormality was observed as a fibrous, transparent membrane in direct contact with both the thoracic wall and the pulmonary lobe and was not accompanied by significant lung fibrosis (Figure 5d).

**Figure 8:**
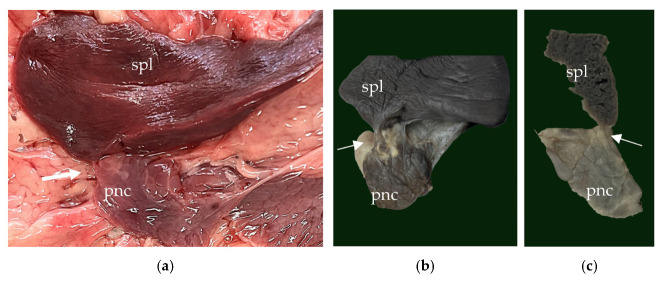
The splenopancreatic fusion: (**a**) the gross aspect of the splenopancreatic fusion (white arrow). (**b**) The formalin-fixed appearance of the fusion (white arrow). (**c**) A longitudinal section of the splenopancreatic fusion (white arrow). spl—spleen, pnc—pancreas.

Besides all the thoracic findings, within the abdominal cavity an extension of the spleen parenchyma into the tail of pancreas was identified macroscopically (Figure 8), which was confirmed histologically as splenopancreatic fusion (Figure 7d).

## 3. Discussion

PTA type I in cats has been documented in the literature in five previous publications [2,3,4,5,6]. These reports describe exclusively domestic short-haired cats, comprising three males and two females. Similarly to our case, the age at diagnosis for the majority of patients ranged from 5 months to 2 years, but the pathology was also identified in a 6-year-old cat [6]. The most commonly reported clinical signs include tachypnea and cyanosis, often exacerbated by stress or exercise. In some cases, clinical signs were noted from an earlier age [6], although for our patient, no information was available from the owner regarding the onset of symptoms.

Except for one case with a continuous 3/6 heart murmur, the remaining patients, including the one in this publication, presented with a 4/6 to 5/6 systolic heart murmur on the left hemithorax. The patients presenting a murmur graded less than 5/6 generally exhibited pink mucous membranes during clinical examinations. This pattern, however, did not apply to our cyanotic patient, who exhibited a 4/6 systolic parasternal murmur audible in both hemithoraxes.

While our case did not show ECG abnormalities, previous reports have documented findings such as sinus rhythm with right axis deviation [5], premature ventricular complexes [6] and atrioventricular dissociation [3]. Because Holter monitoring was not performed, rhythm abnormalities cannot be excluded. The echocardiographic findings in our case were consistent with those described in earlier reports, such as dilation of the right atrium, biventricular hypertrophy, a large ventricular septal defect visible on 2D images [2,3].

Our case presents a rare finding of a persistent truncus arteriosus type I in a cat, associated with a left atrial diverticulum (LAD), a 5 mm smooth-walled outpouching communicating with the atrial lumen, with a preserved histology of the wall. This structural and histological description parallels findings in humans, where LADs are defined as small pouch-like structures with smooth contours, typically less than 1 cm in size and considered anatomical variants [10,15]. Even in humans, left atrial diverticulum (LAD) is a rare malformation [9].

The etiology of LAD in humans is debated, with both congenital origins—arising from developmental weaknesses in the atrial wall—and acquired causes, such as atrial wall damage from disease or surgery, being proposed [16,17,18,19,20]. Given the preserved histological architecture in our case, the congenital hypothesis appears plausible. Furthermore, the myocardial thinning observed aligns with descriptions in human LAD and other cardiac diverticula, such as a feline left ventricular apical diverticulum characterized by myocardial thinning and preserved histological layers [21,22]. Only a single case of a congenital right atrial diverticulum in a kitten was previously reported [23].

The current case of myxomatous degeneration affecting both the mitral and tricuspid valves aligns with known characteristics of myxomatous degenerative valve disease, although within a less common context for cats. In dogs, this condition is the leading cardiac disease, predominantly affecting the mitral valve (~60% of cases), with combined mitral and tricuspid involvement seen in ~30% of cases. Tricuspid valve-only disease is particularly rare (<10%) [24]. While cats can also develop myxomatous degeneration, it is considered an uncommon finding, typically localized to the mitral valve [24,25]. A previous report of myxomatous degeneration in a cat highlighted the rarity of the pathology in cats and provided similar histological findings tp the presented case. In that case, a 1-year-old cat with hypertrophic cardiomyopathy exhibited severe myxomatous degeneration of the mitral valve, characterized by thickening of the spongiosa and fibrous disruption, alongside congenital abnormalities of the tricuspid valve [26].

The segmentation, particularly the multi-segmental structure of the sub arterial papillary muscle and the bifid posterior papillary muscle, deviates from the standard anatomical descriptions provided for both cats and dogs [27,28]. In normal feline anatomy, papillary muscles can occasionally present a divided aspect into two or three lobes or can be accompanied by small accessory reliefs. Although feline cardiac anatomy is similar to that of dogs, their papillary muscles, particularly the sub arterial papillary muscles, tend to be stronger [27].

Hypertrophy of the papillary muscles, such as observed here, has been well-documented in association with hypertrophic cardiomyopathy (HCM) in cats. Studies indicate that enlarged papillary muscles are a consistent feature of feline HCM, often accompanying left ventricular hypertrophy [29,30,31]. Severe papillary muscle enlargement is even considered an echocardiographic characteristic of HCM [32].

Splenopancreatic fusion represents a rare congenital anomaly. This condition, which includes fusion of the pancreatic tail and splenic hilum or the presence of ectopic pancreatic or splenic tissue, is attributed to disturbances in embryogenesis due to the close interaction between the pancreas and spleen within the dorsal mesogastrium during development [33]. While splenopancreatic field abnormalities are well-documented in humans and have been associated with congenital disorders like trisomy 13 [34], reports in veterinary species, including cats, are lacking.

In human medicine, splenopancreatic fusion is typically identified incidentally during imaging or surgical exploration, with few documented radiological findings [34]. Its clinical significance lies in the potential for complications during surgical interventions, such as splenectomy or distal pancreatectomy, underscoring the importance of recognizing this anomaly preoperatively [33].

During this research, the authors were not able to find a classification of nomenclature for the abnormal pleural fusion, and further investigations are required in relation to this matter.

## 4. Conclusions

In conclusion, this case highlights a rare presentation of persistent truncus arteriosus (PTA) type I in a cat, uniquely associated with a left atrial diverticulum (LAD). The findings expand the understanding of LAD in veterinary species and draw parallels to congenital abnormalities documented in human medicine. This case emphasizes the importance of detailed diagnostic evaluations in identifying and characterizing complex congenital anomalies in veterinary cardiology.

## Data Availability

The original contributions presented in this study are included in the article and Supplementary Materials. Further inquiries can be directed to the corresponding author.

## Supplementary Materials

The following supporting information can be downloaded at: https://www.mdpi.com/article/10.3390/ani15060899/s1. Video S1. Right parasternal long axis view from a 2-year-3-month-old domestic shorthair neutered male cat. Contrast echocardiography confirms the presence of the interventricular communication, Video S2. Right parasternal short axis view from a 2-year-3-month-old domestic shorthair neutered male cat underlying flattening of the interventricular septum, hypertrophy of the ventricle walls and the ventricular septal defect, Video S3. Right parasternal long axis view from a 2-year-3-month-old domestic shorthair neutered male cat showing the ventricular septal defect, a severely thickened tricuspid valve, bilateral hypertrophy of the ventricles and right atrial dilation.
